# Total control of fat cells from adipogenesis to apoptosis using a xanthene analog

**DOI:** 10.1371/journal.pone.0179158

**Published:** 2017-06-05

**Authors:** Ching-Hsuan Tung, Myung Shin Han, Jianjun Qi

**Affiliations:** 1 Molecular Imaging Innovations Institute, Department of Radiology, Weill Cornell Medicine, New York, NY, United States of America; 2 Department of Translational Imaging, Houston Methodist Research Institute, Houston, TX, United States of America; INIA, SPAIN

## Abstract

Overcrowded adipocytes secrete excess adipokines and cytokines under stress, which results in a deregulated metabolism. This negative response to stress increases the possibility of obesity and several of its associated diseases, such as cancer and atherosclerosis. Therefore, a reduction in the number of adipocytes may be a rational strategy to relieve the undesired expansion of adipose tissue. A newly synthesized xanthene analog, MI-401, was found to have two distinct effects on the regulation of the adipocyte’s life cycle. MI-401 efficiently down regulated the expression of transcription factors, PPARγ and C/EBPα, and lipogenesis proteins, FAS and FABP4. This down regulation resulted in the inhibition of adipogenesis. Without newly differentiated adipocytes, the total number of adipocytes will not increase. In addition to this inhibitory effect, MI-401 was able to actively kill mature adipocytes. It specifically triggered apoptosis in adipocytes at low micro molar concentration and spared preadipocytes and fibroblasts. These dual functionalities make MI-401 an effective agent in the regulation of the birth and death of adipocytes.

## Introduction

Adipocytes are the primary cell type in fat tissue. These cells collect excess triglycerides and place them into lipid droplets as energy depots. The total adipose tissue mass depends on the number and size of the adipocytes. During childhood and adolescence, adipocytes arise through adipogenesis from fibroblast-like progenitor cells, and the initial adipocyte generation process becomes static post adolescence.[1] Under normal metabolic conditions, adipocytes have a mean lifespan of 10 years.[2] Only 10% of all adipocytes undergo a yearly renewal process, which is tightly balanced between the adipogenesis of preadipocytes and the apoptosis of adipocytes. However, recent studies suggested that a prolonged period of obesity may cause the body to recruit new preadipocytes and stimulate their differentiation into mature adipocytes, increasing the number of total adipocytes.[3, 4] As the number increases, the continuous deposit of triglycerides further cause adipocytes to grow in size.[5] The overexpansion of adipose tissue yields to severely dysfunctional adipocytes that secrete adipokines and cytokines, such as leptin and adiponectin, and cause alterations to their normal metabolism.[6] Obesity, therefore, has been linked to many chronic diseases and metabolic disorders including diabetes[7], atherosclerosis[8, 9] and cancer.[10, 11]

Control over the unhealthy expansion of adipose tissue would pose a significant benefit to the management of obesity and its associated diseases. Current anti-obesity drugs are primarily based on appetite suppression and reduction in fat uptake.[7, 12] The treatments are able to change the size of adipocytes, which result in marked weight loss, but the total number of adipocytes remains the same.[1] Once the treatment stops, the contracted adipocytes expand to regain their original size. Therefore, although weight management is an excellent step towards obtaining a healthy metabolism, it may not be the best solution for all obese adults. A reduction in the total number of adipocytes, by cutting their supply or reducing their inventory, may be a more effective strategy in regulating the expansion of unhealthy fat tissue.

Adipocytes are terminally differentiated cells. When induced by adipogenic stimuli, the committed preadipocytes undergo mitotic clonal expansions and become adipocytes. Inhibitors that block adipogenesis, both natural products[13–18] and synthetic molecules[19–22], have been proposed to prevent the differentiation of these preadipocytes. Furthermore, reagents that can push mature adipocytes into apoptosis have also been suggested as a possible strategy in controlling obesity.[23, 24] Unfortunately, possible drug candidates have been restricted to natural products and plant extracts due to an inadequate understanding of the adipocyte’s apoptotic mechanism.[25–32] A few natural molecules and combinations of those molecules have been identified as apoptosis triggers and differentiation inhibitors, but their efficacy is mild. Often, a high concentration, greater than 100 μM, is needed to obtain an appreciable effect in culture.[25–28, 31–34] In 2015, sodium deoxycholate (SD) was approved by the FDA to reduce the unwanted submental fat, a very different compound from those previously available.[35–37] SD acts like a detergent, causing adipolysis (or adipocytolysis) when injected directly into the area with extra fat tissue.[36, 38] SD lyses the adipocyte’s membrane, which is deficient in cell associated proteins, resulting in necrosis.[37, 38] The usage of SD has been limited to the removal of local fat tissue because the required active dose is high, 2 mg/ml (~ 5 mM) and 0.2 ml/cm^2^.[37]

**Fig 1:**
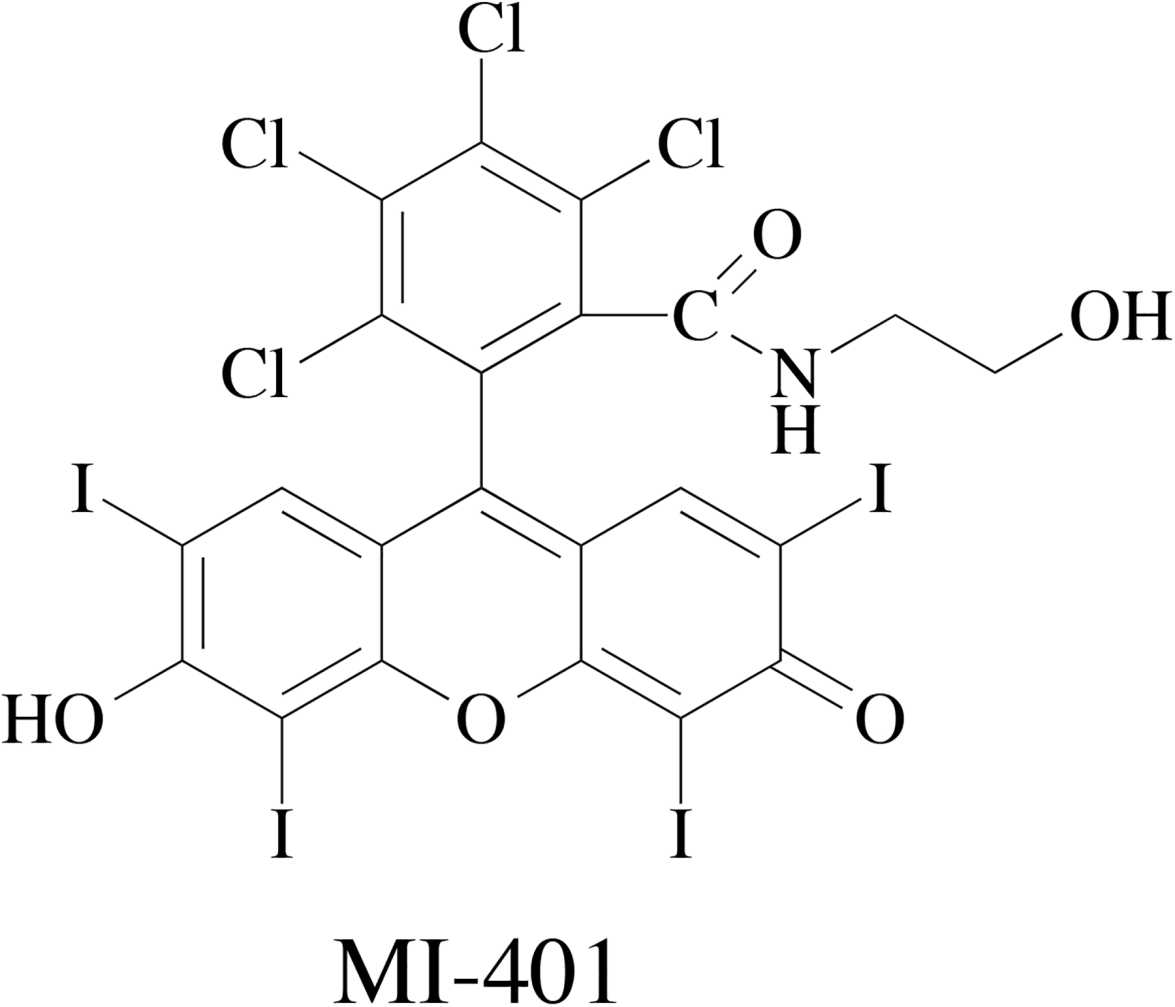
The chemical structure of MI-401.

Planar tricyclic oxygen containing xanthene molecules are known to have various bioactivities.[39–42] For example, a fluorescent xanthene dye, Rose Bengal, is an effective photosensitizer for photodynamic therapy.[43] Previously, we reported a membrane sensitizing Rose Bengal derivative, which killed cancer cells by immediate membrane lysis when insonated by ultrasound,.[44] Several derivatives were later synthesized to study their membrane sensitizing property in cancer cells. Since adipocyte contains a unique cell membrane, one synthesized molecule, MI-401 (2, 3, 4, 5- tetrachloro- 6- (6- hydroxy- 2, 4, 5, 7- tetraiodo- 3- oxo- 3H- xanthen- 9- yl)—N- (2- hydroxyethyl) -benzamide; Fig 1), was tested with adipocytes. Unexpectedly, it was found that MI-401 regulates the adipocyte’s life cycle in two ways. MI-401 effectively inhibits the adipogenesis process by differentiation arrest, IC_50_ = 3 μM, and kills mature adipocytes through the induction of apoptosis, EC_50_ = 5 μM.

## Materials and methods

### Reagents and cells

Sodium deoxycholate (SD), 4,5,6,7-tetrachloro-2',4',5',7'-tetraiodofluorescein, N,N,N′,N′-Tetramethyl-O-(1H-benzotriazol-1-yl)uronium hexafluorophosphate, O-(Benzotriazol-1-yl)-N,N,N′,N′-tetramethyluronium hexafluorophosphate (HBTU), diisopropoyl ethyl amine (DIEPA), and Dimethyl sulfoxide (DMSO) were obtained from Sigma-Aldrich (St. Louis, MO, USA). All other solvents, including dimethyl formamide (DMF), dichloromethane (DCM), and methanol (MeOH), were purchased from Thermo Fisher (Waltham, MA).

The triglyceride quantification kit AdipoRed^TM^ was obtained from Lonza Walkersville (Walkersville, MD). CellMask^TM^ Plasma Membrane Stain with DAPI, and LipidTox^TM^ DeepRed neutral lipid stain were purchased from Thermo Fisher. The LumiGLO® reagent used in Western blot studies, and antibodies against the fatty acid-binding protein4 (FABP4), peroxisome proliferator-activated receptor γ (PPARγ), CCAAT element binding protein α (C/EBPα), fatty acid synthase (FAS) and β-Actin were obtained from Cell Signaling Technology (Beverly, MA, USA). The BCA protein assay kit and M-PER were purchased from PIERCE (Rockford, IL, USA). CellTiter 96® AQ_ueous_ One solution (MTS) Cell Proliferation Assay kit, ApoOne^®^ Homogeneous Caspase 3/7 assay kit and CytoTox-One^TM^ Homogeneous Membrane Integrity Assay kit were from Promega (Madison, WI, USA).

3T3-L1 preadipocytes (passage 7 to 8) and culture media were obtained from Zen-Bio (Research Triangle Park, NC, USA). Only passage 8 to passage 11 were used in the study. NIH3T3 fibroblast cells were obtained from ATCC (Manassas, VA, USA) and maintained in a DMEM medium with 10% FBS at 37°C and 5% CO_2._

### Synthesis of MI-401 (2,3,4,5-tetrachloro-6-(6-hydroxy-2,4,5,7-tetraiodo-3-oxo-3H-xanthen-9-yl)-N-(2-hydroxyethyl)-benzamide)

MI-401 was prepared following a described procedure.[44] Briefly, 4,5,6,7-tetrachloro-2',4',5',7'-tetraiodofluorescein (0.5 mmol) was activated by HBTU (0.5 mmol) in DIEPA/DMF (2/3, 5 ml) and stirred at room temperature (RT) for 4 hours. 2-Aminoethanol (1.5 mmol) was then added and reacted overnight at RT. The solvent was removed under vacuum. The residue was extracted with DCM and washed with brine, dried over anhydrous sodium sulfate and concentrated. It was then purified by a silica gel column, and eluted with DCM, DCM/MeOH = 10/0.5 and 10/1 (V/V), to give a pale yellow solid product (yield 31%).

### General culture and differentiation conditions for 3T3-L1 cells

3T3-L1 preadipocytes were grown in 3T3-L1 Preadipocyte Medium (PM-1-L1) with 5% CO_2_ at 37°C for two to three days to reach confluence. The medium was replaced with a 3T3-L1 Differentiation Medium (DM-2-L1) to stimulate differentiation. After 3 days in DM-2-L1, the differentiated cells were maturated with 3T3-L1 Adipocyte Maintenance Medium (AM-1-L1) for an additional 3 days. This general protocol was used in all experiments, but adjusted with various treatments. The detailed experimental scheme is described in each figure.

### MI-401 Stock solution preparation

MI-401 was dissolved in pure DMSO (Sigma) to obtain 1 to 20 mM stock solutions. Prior to each cell experiment, the MI-401 stock solution was diluted to the desired concentration using cell culture medium. After dilution, the final DMSO concentration was no higher than 1%. The DMSO solution (1%) presented no significant toxicity to the tested cells.

### MTS assay for cell viability and EC_50_

Cells (3,000/well) were seeded in a 96-multiwell plate (Corning Costar) with complete medium (100μL per well), and incubated at 37°C, with 5% CO_2_. The cells were then treated with various concentrations of MI-401 or SD (1–400 μM) for the indicated time periods. The MTS assays in a 96-well plate were done with a CellTiter 96® AQ_ueous_ reagent (20 μl, Promega) in a culture medium (100 μl). The plates were incubated at 37°C for 4 h. Absorbance was measured at 490 nm using a microplate reader (Infinite M1000 Pro, Tecan, Männedorf, Switzerland). The experiments were done in at least triplicate.

### Measurement of lipid accumulation

The triglyceride accumulation was quantitated after the cells underwent treatments with different amounts of MI-401 for 1 or 2 days. The cells were rinsed with PBS prior to the commencement of the assay. An AdipoRed^TM^ solution (30 μL) was added to each well, homogenized by pipetting, and then incubated for 10–15 min at RT. The amount of triglyceride was measured using a fluorescent plate reader (Infinite M1000 Pro, Tecan) with a 485 nm excitation and a 572 nm emission. The experiments were done in triplicate.

### Lipid stain for fluorescence microscopy

The cells were washed twice with the phosphate-buffered saline (PBS), fixed with 3.7% formaldehyde solution for 10 minutes at RT, and then rinsed gently 2–3 times with a PBS buffer to remove residual formaldehyde. LipidTox^TM^ Deep Red stain solution (1:200 in a buffer) was added to the wells and incubated at RT for 30 minutes before fluorescence imaging using a Cy5 filter (ex = 650 nm, em = 670 nm).

### Plasma membrane and DAPI staining for Cell death image

The plasma membrane and nucleus were stained using a CellMask^TM^ Plasma Membrane Stain with DAPI (Thermo Fisher) in accordance with the manufacture’s protocols for 5–10 min at 37°C. The cells were imaged with a fluorescent microscope (Evos FL, Thermo Fisher) using GFP and DAPI filters.

### Cell death mechanism study

Caspase3/7 activity assay was done with the ApoOne Homogeneous Caspase 3/7 assay (Promega) following the manufacturer’s instructions. Matured adipocytes were treated with SD (50 μM) or MI-401 (10 μM) in 100μL of AM-1-L1 (3T3-L1 Adipocyte Maintenance Medium) and then Apo-One Caspase-3/7 Reagent (100 μL) was added to each well. These wells were gently mixed using a plate shaker at 300 rpm for 30 sec. The fluorescence reporting the caspase activity was monitored using a fluorescence plate reader (Infinite M1000 Pro, Tecan) for 18 hours at RT with a 499 nm excitation and 521 nm emission. The experiments were done in triplicate.

LDH release was assayed using the CytoTox-ONE kit (Promega) following the manufacturer’s instructions. The cells, in a 96-well plate, were added with a CytoTox-ONE Reagent (100 μL). The plate was gently shaken at 300 rpm for 30 sec, incubated at 22°C for 10 min, and then stopped by a stopping solution (50μL). The fluorescence was measured using a fluorescence plate reader (Infinite M1000 Pro, Tecan) with a 560 nm excitation and a 590 nm emission. Percent cytotoxicity was calculated following the manuscript’s instructions. Percent cytotoxicity = 100 x (Experimental–Culture medium background)/(Maximum LDH release–Culture medium background). The experiments were done in triplicate.

### Preparation of cell lysates and Western blotting

After treatments, the cells were washed twice with PBS and lysed with M-PER (PIERCE) on ice for 5 min. The lysates were centrifuged at 14,000x g for 5 min, and the supernatants were collected. Protein concentrations were determined using a BCA protein assay kit (PIERCE). The proteins were separated by SDS-PAGE (4~12%) and transferred to nitrocellulose membranes. The membranes were washed with 0.05% (vol/vol) Tween 20 in PBS, followed by blocking with 5% (wt/vol) non-fat dried milk. The membranes were incubated overnight with antibodies specific for C/EBPα (1:1000), PPARγ (1:1000), FAS (1:1000), and FABP4 (1:1000) and β-actin (1:5000) at 4°C. The membranes were then washed with PBS and exposed to secondary antibodies coupled with horseradish peroxidase (Anti-rabbit IgG, HRP-linked antibody, Cell Signaling) for 2 hours at RT. The membranes were washed three times for 5 min with PBS at RT. Immunoreactivities were detected by an enhanced LumiGLO® reagent (Cell Signaling) and documented using the F-Pro imaging system (Bruker, Billerica, MA).

### RNA isolation and qPCR analysis

In a separate set of cells, 3T3-L1 cells were treated using the same method for the Western blot analysis. At the designated time points, the total RNA from the control or MI-401 treated 3T3-L1 cells was extracted using a Direct-zol RNA MicroPrep kit with Trizol® reagent (Zymo) following the manufacturer’s instruction. Each RNA sample (1μg) was reverse transcribed to cDNA using iScript Reverse Transcription Supermix (Bio-Rad, Hercules, CA).[45] For RT-qPCR, 2 μl of a 1:10 dilution of the cDNA was used per well in a 10 μl reaction, along with 1 μl of primer set (300 nM) and 5 μl of iTaq^TM^ Universal SYBR Green Supermix (Bio-Rad). The reactions were run in triplicate at least on a StepOnePlus Real-Time PCR Detection System (Applied Biosystems, Foster City, CA) with the following protocol: 95°C (2 min), 40 cycles of 95°C (15 s) and 60°C (60 s). All primers were purchased from Integrated DNA Technologies (Coralville, IA). 18s rRNA and β-Actin were used as reference housekeeping genes for normalization and quantification using the 2-ΔΔC(t) method [46]. The primer sequences used were [20, 30]: FABP4: sense 5’-AGTGGGAGTGGGCTTTGCCA-3’ and antisense 5’-GGGCCCCGCCATCTAGGGTTA-3’; C/EBPα: sense, 5’-TTACAACAGGCCAGGTTTCC-3’ and antisense 5’-CTCTGGGATGGATCGATTGT-3’; FAS: sense 5’-TTGCTGGCACTACAGAATGC-3’ and antisense 5’-AACAGCCTCAGAGCGACAAT-3’; PPARγ: sense 5’-GGTGAAACTCTGGGAGATTC-3’ and antisense 5’-CAACCATTGGGTCAGCTCTC-3’; β-Actin: sense 5’-GGTGAAGGTCGGTGTGAACG-3’ and antisense 5’-GGTAGGAACACGGAAGGCCA-3’; 18s rRNA; sense 5’-CTTAGAGGGACAAGTGGCG-3’ and antisense 5’-ACGCTGAGCCAGTCAGTGTA-3’.

### Statistical analysis

All values are presented as means ± standard deviation. P-values <0.05 were considered statistically significant. Statistical significance was determined using a student’s t-test. All analyses were performed using the GraphPad Prism program (version 7, La Jolla, CA, USA).

## Results and discussion

### MI-401 killed mature adipocytes at low μM concentration

**Fig 2:**
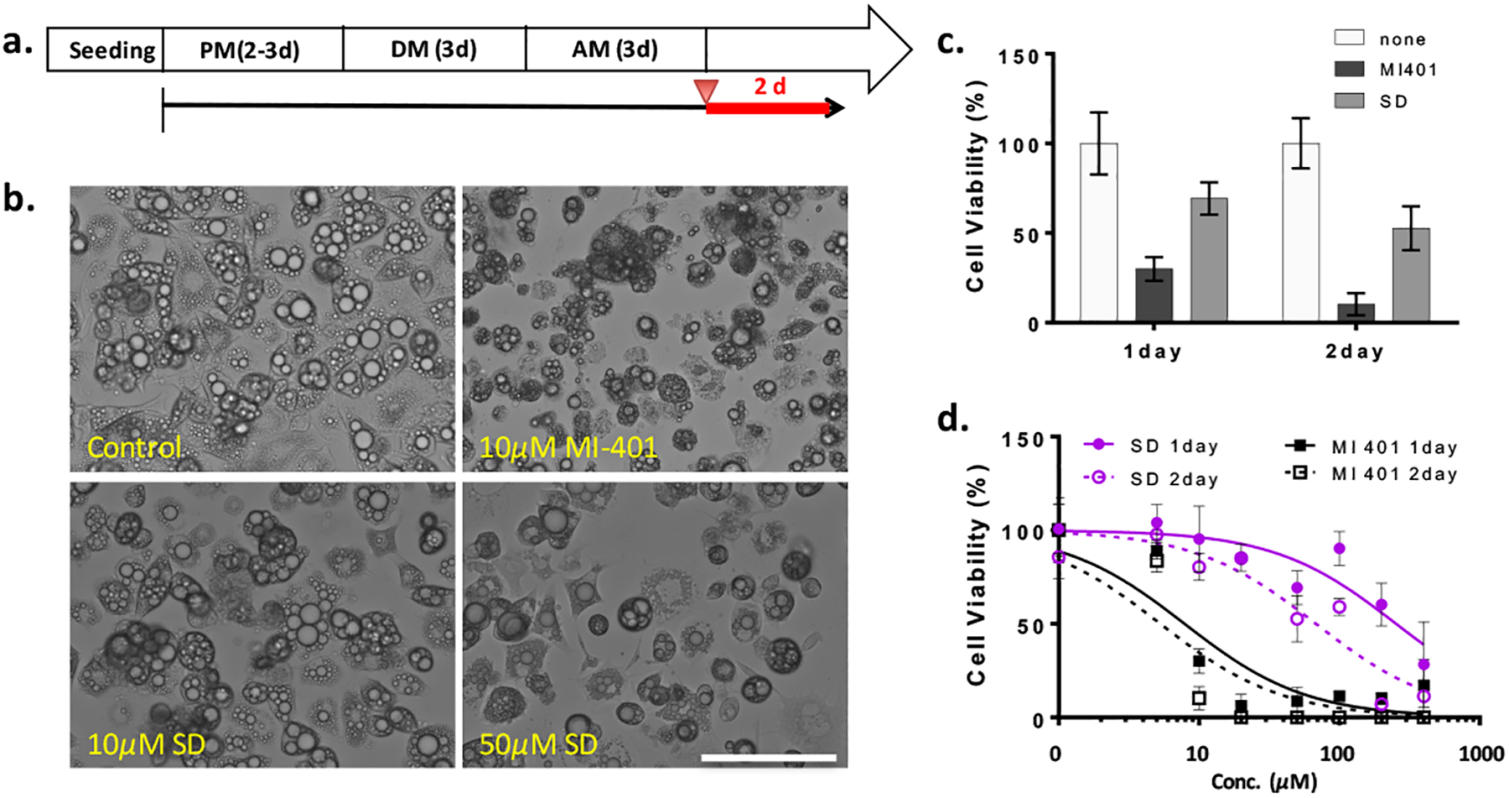
Effect of SD and MI-401 on mature adipocytes. (A) 3T3-L1 cells were cultured in preadipocyte medium (PM), differentiated in differentiation medium (DM), and then maturated in adipocyte maintenance medium (AM). Drugs were added at day 3 after maturation (indicated by red arrowhead). The treatments lasted for 1 or 2 days (Red line). (B) Representative images of mature 3T3-L1 cells treated with MI-401 (10 μM) or SD (10 or 50 μM). Scale bar = 100 μm. (C) Comparison of cell viability of MI-401 (10 μM) and SD (50 μM) treated adipocytes. Data is presented as mean ± standard deviation (n ≥ 3). (D) Viability of cultured adipocytes treated with SD or MI-401. The determined EC_50_ for MI-401 after 1-day or 2-day of treatment was 7.9 or 5.3 μM, respectively; and for SD after 1-day or 2-day of treatment was 253.8 or 52.8 μM, respectively. Data is presented as mean ± standard deviation (n ≥ 3).

SD is currently the only FDA approved drug for the treatment of excess local fat tissue; thus, the cell-killing efficacy of MI-401 was compared to SD using mature 3T3-L1 adipocytes as a model. 3T3-L1 preadipocytes were grown in an adipocyte differentiation medium for 3 days. These cells were then maturated in an adipocyte maintenance medium for an additional 3 days. Once matured, MI-401 and SD were added to the adipocyte’s maintenance medium. The cells were then analyzed one or two days after treatments (Fig 2A). Microscopic examination at day 2 found significant morphological changes in the MI-401 (10 μM) treated cells, which were small, dense, and rough (Fig 2B). In contrast, no difference was observed between the untreated mature adipocytes and the SD (10 μM) treated cells. The morphology was clearly different once the administered concentration of SD was raised to 50 μM. The viability of the adipocytes with MI-401 (10 μM) was down to 30% on day 1 and 10% on day 2 (Fig 2C); while SD (50 μM) treated groups still had 70% or 53% of viable cells one or two days after treatment. Subsequent systematic viability studies showed the EC_50_ of MI-401 and SD at post treatment day 2 to be 5.3 and 53 μM, respectively (Fig 2D). The EC_50_ of MI-401 on day 1 was 7.9 μM.

### MI-401 triggered apoptosis in mature adipocytes

**Fig 3:**
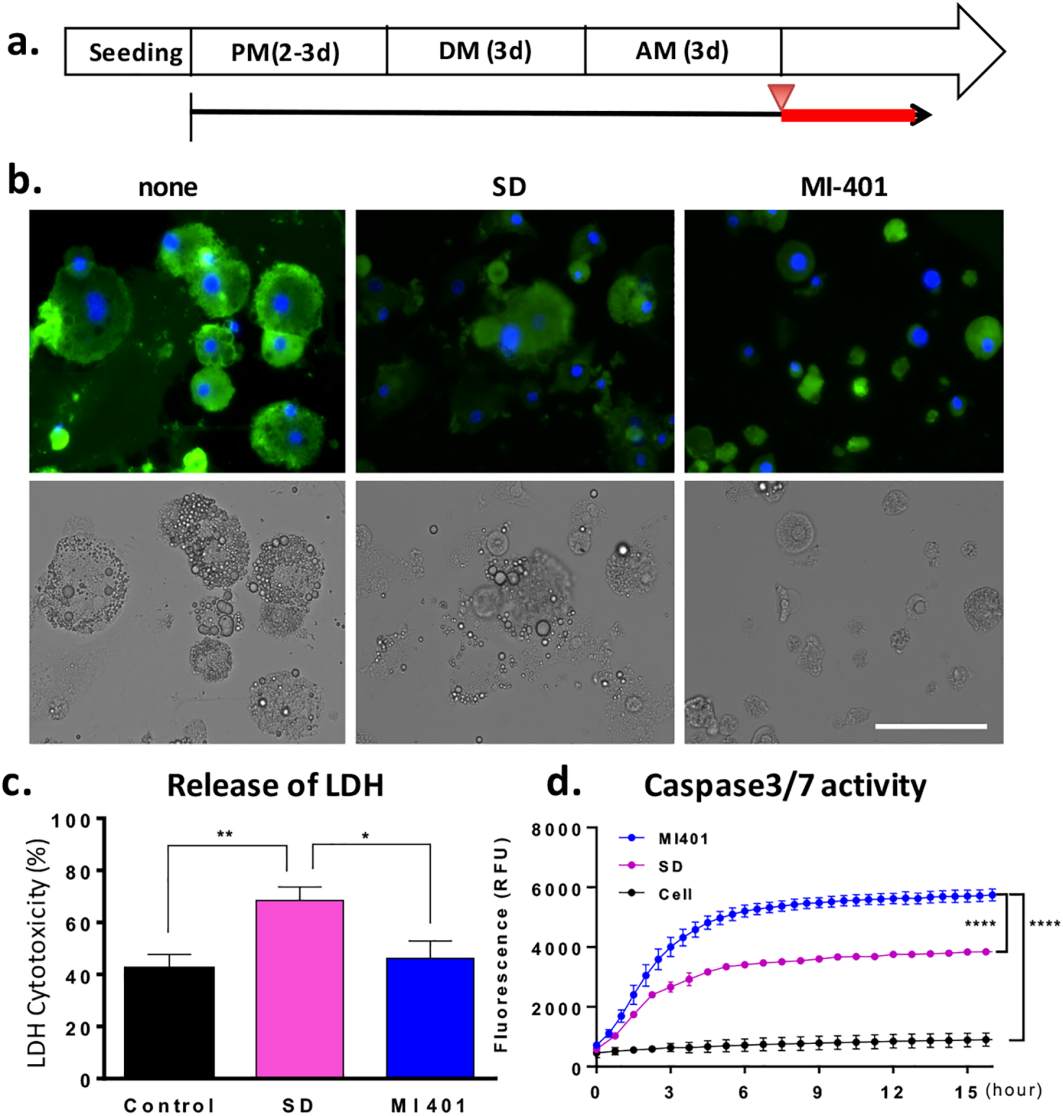
Death mechanism of SD and MI-401 treated adipocytes. (A) 3T3-L1 cells were cultured in preadipocyte medium (PM), differentiated in differentiation medium (DM), and then maturated in adipocyte maintenance medium (AM). SD and MI-401 was added at day 3 after maturation (red arrowhead). The drug treatments lasted 2 days (Red line). (B) Representative images of mature 3T3-L1 cells treated with MI-401 (10 μM) or SD (50 μM). The cell membrane was stained with CellMask^TM^ Plasma Membrane Stain (Green) and the nucleus was stained with DAPI (Blue). Scale bar = 100 μm. (C) Quantitative analysis of necrosis associated LDH release 4 hrs after SD (50 μM) or MI-401 (10 μM) treatment. The LDH released from the total lysed cells was set as 100% LDH cytotoxicity. The untreated adipocytes were used as the negative control. Data is presented as mean ± standard deviation (n = 3). (Unpaired t-test, ** P < 0.005, * P < 0.05) (D) Time course of caspase 3 and 7 activity after SD treatments (50 μM) or MI-401 (10 μM). The fluorescence signal representing caspase 3/7 activity was determined over 18 hours using a fluorescence plate reader. Data is presented as mean ± standard deviation (n = 3). (Paired t-test, **** P ≤ 0.001).

When compared to the clinically approved SD, MI-401 proved to be approximately 10 times more effective in killing mature adipocytes. In addition to its stronger killing efficacy, a difference in the cell morphology between the treated groups was observed. This difference suggests that the treated cells may have died through different death pathways (Fig 2B). To better visualize the cell’s morphology, cells were co-stained with CellMast^TM^ Plasma membrane stains and DAPI nuclear stains 2 days after treatment (Fig 3A and 3B). When compared to untreated mature adipocytes, SD treated cells are about the same in size, but possess a substantially lower membrane fluorescence intensity. This lowered fluorescence suggests a partial solubilization of the membrane (Fig 3B). Additionally, upon further observation, the lipid droplets appear to have diminished from the cytoplasm. In contrast, MI-401 treated cells have become smaller. Apoptotic characteristics such as cytoplasm shrinkage, nucleus condensation, cell debris and the disappearance of lipid droplets were observed.

SD is known to destroy adipocytes by breaking down or solubilizing cell membranes[37, 47]; therefore, a lactate dehydrogenase (LDH) release assay was selected to study the integrity of the plasma membranes post treatment. As expected, SD treated cells exhibited a significant release of cytosolic LDH which was ~70% over the background value. MI-401 treated cells, however, were only about 25% higher (Fig 3C). The results of this assay suggested that MI-401 does not affect adipocytes by lysing the plasma membrane as SD does. An apoptosis fluorescence assay was then conducted to determine the activity of triggered apoptotic enzymes, caspase 3 and caspase 7. The caspases’ activity of the MI-401 treated group steadily increased over the measuring period, and the determined signal was significantly higher than that of the SD treated group (Fig 3D). These experimental results further indicate that MI-401 is an effective apoptosis initiator.

### MI-401 inhibited the differentiation of preadipocytes

**Fig 4:**
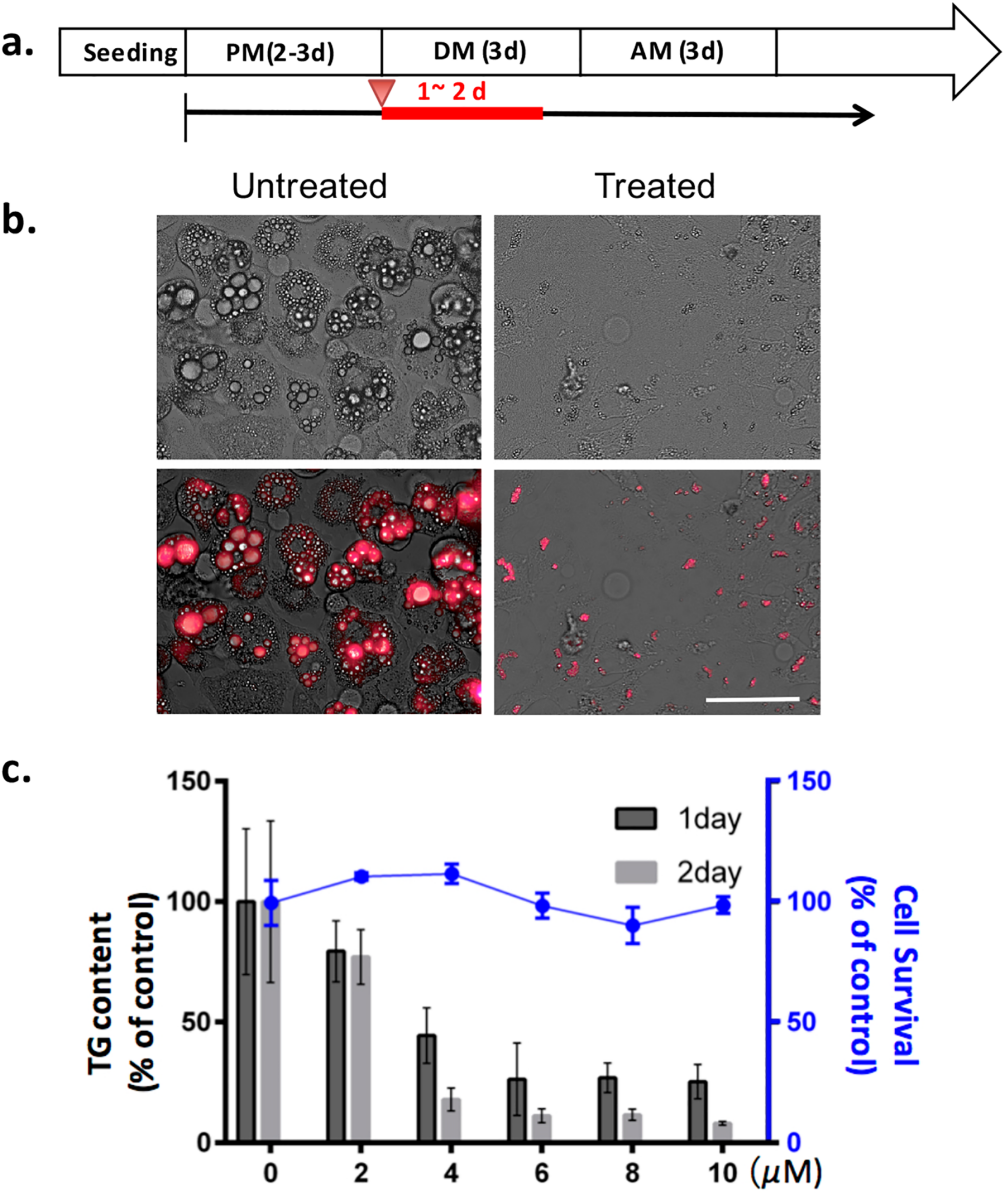
Inhibitory effect of MI-401 during the early stage of adipogenic differentiation. (A) 3T3-L1 cells were cultured in preadipocyte medium (PM) for 2–3 days, and then treated with MI-401 in the differentiation medium (DM) for the indicated time periods (Red line). The cells were continuously cultured in an adipocyte maintenance medium (AM) for an additional 3 days post differentiation. (B) Representative images of the lipid stain in the untreated and treated 3T3-L1 adipocytes. The cells were stained with HCS Lipdox^TM^ lipid stain prior to imaging. Scale bar = 100 μm. (C) Quantitative analysis of triglyceride accumulation with MI-401 treatment (black bars and left Y-axis) and cell viability after treatments (blue line and right Y-axis). Data is presented as mean ± standard deviation (n ≥ 3). Based on triglyceride content, the IC_50_ of MI-401 with 1-day or 2-day treatment was 3.2 and 2.5 μM, respectively.

MI-401 was then checked for its inhibition potential of adipogenesis. 3T3-L1 preadipocytes were seeded and grown to confluence in a preadipocyte maintenance medium for 2–3 days. Thereafter, the cells were cultured in a differentiation medium containing different amounts of MI-401 for one or two days (Fig 4A). Following a 3-day differentiation period, the cells were further cultured in an adipocyte maintenance medium for an additional 3 days and analyzed for their lipid contents. As seen in Fig 4B, the fully differentiated 3T3-L1 adipocytes were rich in large lipid droplets. A two-day incubation with 10 μM of MI-401 in a differentiation medium, almost completely stopped the occurrence of lipogenesis (Fig 4B) in the treated cells. A triglyceride (TG) quantification assay was preformed to determine its IC_50_ after 1-day and 2-day treatments, which resulted in 3.2 and 2.5 μM, respectively (Fig 4C). Importantly, under testing conditions, this differentiation-arresting drug was found to be non-toxic to differentiating preadipocytes (Fig 4C).

### MI-401 blocked the early steps of adipogenesis

**Fig 5:**
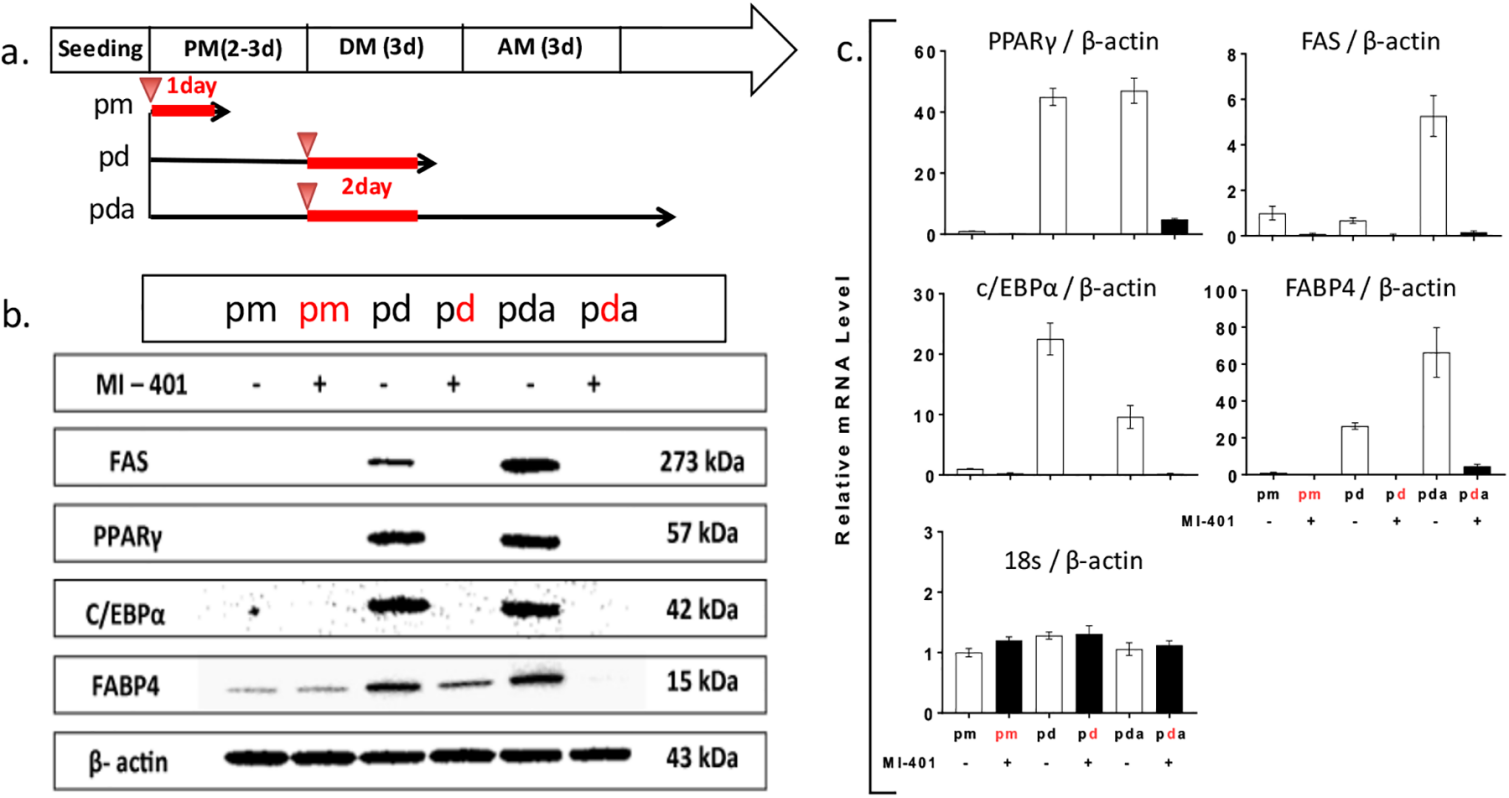
Effect of MI-401 on adipogenic gene expression in different stages of growth. (A) MI-401 (10 μM) was added to the preadipocyte maintenance medium (pm group), or differentiation medium (pd group). The cells were harvested right after the treatment. In the third set of cells (pda group), MI-401 was added to the differentiation medium for 2 days, and then the cells were cultured in the adipocyte maintenance medium for additional 3 days. The red letter indicates the drug treated groups, and the red line indicates the duration of the treatment. (B) Representative Western blot analysis showing the effect of MI-401 on PPARγ, C/EBPα, FAS and FABP4. (C). Relative mRNA level of PPARγ, C/EBPα, FAS and FABP4. Results were expressed relative to untreated cells after normalized to β-actin. Data are expressed as the mean ± standard deviation. Black bar = with MI-401, empty bar = without MI-401.

Following the observation of a strong suppression of lipid deposition during the differentiation, the regulation of four key factors, including two transcription factors: peroxisome proliferator-activated receptor-gamma (PPARγ) and CCAAT/enhancer-binding protein alpha (C/EBPα), and two lipogenesis players: fatty acid synthase (FAS) and fatty acid binding protein 4 (FABP4),[48] that participated in the adipogenic progression were analyzed by Western blotting and qPCR to understand the possible mechanism of adipogenesis arrest. MI-401 (10 μM) was added to the preadipocyte phase or to the differentiation phase. The cells were collected at different stages and subjected to analysis (Fig 5A). The Western results showed that all four factors had low values at the normal preadipocytes stage; however, when the cells began differentiating, the transcription factors, PPARγ and C/EBPα, were highly upregulated (Fig 5B). FAS and FABP4 were also observed to be upregulated but to a lesser extent. After the cells’ completion of their differentiations, FAS and FABP4, which participated in lipid synthesis, were further expressed for lipid synthesis and deposition. No difference of expression was observed when preadipocytes were treated with MI-401. Nonetheless, when MI-401 was included in the differentiation medium, the expressions of PPARγ, C/EBPα, and FAS by the differentiating preadipocytes were completely blocked. The expression of FABP4 was also significantly down regulated. The suppression of these factors was maintained even after the media was switched to an adipocyte maintenance medium. These results suggest that once the differentiation process is arrested, it cannot be quickly recovered.

To further confirm the regulation of these four factors, RT-PCR was used to quantitate the relative mRNA in all tested conditions (Fig 5C). It was found that the mRNA results corroborated with the Western protein analysis. High mRNA levels of PPARγ and C/EBPα transcription factors were observed in normal differentiation condition and maturation condition, while high mRNA levels of FAS and FABP4 lipogenesis factors were increased in the normal maturation stage. As expected, MI-401 treatment effectively suppressed all four factors at the transcriptional level.

### MI-401 is less cytotoxic to preadipocytes and fibroblast

**Fig 6:**
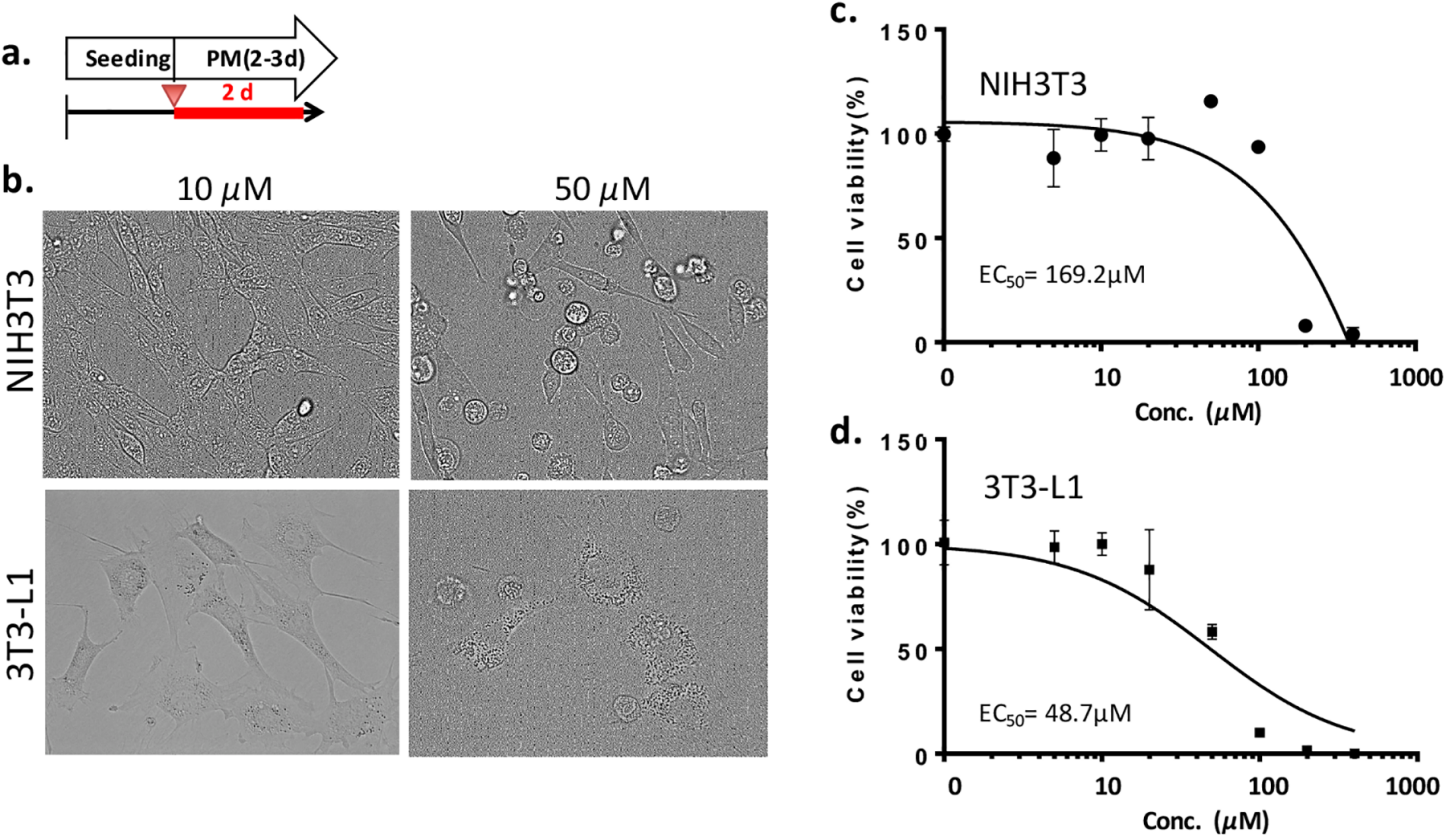
Cytotoxicity of MI-401 on 3T3-L1 preadipocytes and NIH3T3 fibroblast. (A) After seeding, 3T3-L1 preadipocytes were treated with MI-401 (red arrowhead) in a preadipocyte maintenance media (PM) for 2 days (red line). NIH3T3 cells were also treated with MI-401 but in DMEM medium with 10% FBS. (B) Representative images MI-401 (10 or 50 μM) treated 3T3-L1 or NIH-3T3. The NIH-3T3 fibroblasts were healthy at both conditions, but round 3T3-L1 preadipocytes were seen after treated with 50 μM of MI-401. (C) Quantitative analysis of cell viability of NIH-3T3 fibroblast with MI-401. The EC_50_ at day 2 was 169.2 μM. Data are presented as mean ± standard deviation (n = 3). (D) Quantitative analysis of cell viability of 3T3-L1 preadipocytes with MI-401. The EC_50_ at day 2 was 48.7 μM. Data is presented as mean ± standard deviation (n = 3).

MI-401 has proven its efficiency in inhibiting adipogenesis. Subsequent studies were done to understand the extent of its cytotoxicity in preadipocytes and normal fibroblasts. To determine it’s EC_50_, 3T3-L1 preadipocytes were seeded and cultured for two days in a preadipocyte maintenance medium together with various amounts of MI-401 (Fig 6A). Fibroblasts (NIH3T3) were also treated with MI-401 in its optimal culture condition. Both cell types remained unharmed and healthy when exposed to a low concentration of MI-401 (10μM). However, the 3T3-L1 preadipocytes were negatively affected in a 50 μM concentration, while NIH3T3 cells retained their health (Fig 6B). The EC_50_ for 3T3-L1 preadipocyte and NIH3T3 cells on day 2 was determined to be 49 and 169 μM, respectively (Fig 6C and 6D).

### MI-401 is a bifunctional drug to control adipocytes

**Fig 7:**
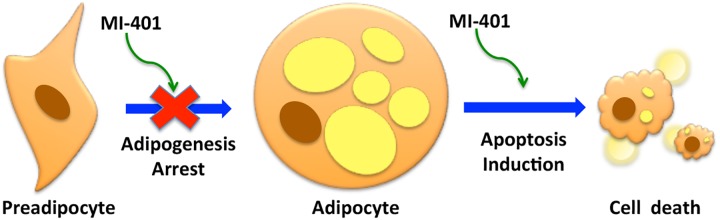
The schematic diagram of the dual functionality of MI-401 to differentiating and matured fat cells. MI-401 inhibits the adipogenesis of preadipocytes, and stimulates apoptosis in adipocytes.

The above results suggest that MI-401 may be useful as a bi-functional drug for the management of adipocytes (Fig 7). MI-401 has proven to efficiently kill mature adipocytes by initiating the death pathway. Unlike SD, which kills mature adipocytes through cellular membrane lysis induced necrosis, MI-401 acts as an apoptosis inducer and is about 10 times more effective in killing mature adipocytes. When in contact with lean preadipocytes, MI-401 inhibits the adipogenesis process completely. It inhibits the expression of transcription factors C/EBPα and PPARγ, which are key players in mid-phase adipocyte differentiations.[48] Subsequent analyses have indicated that this inhibition tends to occur at a very early phase of differentiation. During normal adipogenesis, the coordination of PPARγ with the C/EBP transcription factor further drive the expression of other adipocyte-specific genes. This includes FABP4, FAS, and several other proteins that participate in the lipogenesis process, which is the final phase of their differentiation into mature adipocytes.[48] The early suppression of C/EBPα and PPARγ by MI-401 has regulated the downstream expression of FABP4 and FAS, which has resulted in the complete arrest of adipogenesis. MI-401 inhibits the differentiation of preadipocytes, but had no effect on its proliferation. When compared to the compounds reported in literature that inhibit adipogenesis or/and induce apoptosis,[12, 16, 23] MI-401 is the only compound capable of acting at low μM concentrations and be non-toxic to both preadipocytes and normal fibroblasts. \

The EC_50_ of MI-401 for mature adipocytes, preadipocytes, and normal fibroblasts is 5, 49 and 169 μM, respectively. This differential cytotoxicity is extremely beneficial in designing a selective treatment that will not harm normal cells. Based on the unique structure of MI-401, the fundamental differences of the tested cells, and the observed results, it is postulated that MI-401 might have a direct interaction with the fragile lipid droplet membranes. Adipocytes treated with MI-401 lost their lipid droplets quickly, resulting in a sudden burst of triglycerides and other contents within cytoplasm that triggered the cell death process. Conversely, the lean preadipocytes and fibroblasts which have few lipid droplets were more resistant to the treatment. However, this speculation remains to be validated.

## Conclusion

In summary, MI-401, a newly synthesized molecule with no known biological activity, is a possible drug candidate for the management of excess adipose tissue. MI-401 is an effectual adipogenesis inhibitor with an IC_50_ of 3 μM, and a potent adipocyte killer with an EC_50_ of 5 μM. Like the clinically approved adipolytic SD for mesotherapy, MI-401 could also be potentially applied to the mesoderm to treat local fat tissues. Due to its low EC_50_ to adipocytes, a significantly lower dose is needed to achieve a similar effect. MI-401 is structurally different from other reported analogs and therefore, may provide a new direction for drug development. Further modification and optimization of MI-401 may lead to a new way of controlling fat tissue and treating obesity.
